# Comparison and agreement between venous and arterial gas analysis in cardiopulmonary patients in Kashmir valley of the Indian subcontinent

**DOI:** 10.4103/1817-1737.74274

**Published:** 2011

**Authors:** Parvaiz A. Koul, Umar Hafiz Khan, Abdul Ahad Wani, Rafiqa Eachkoti, Rafi A. Jan, Sanaullah Shah, Zarka Masoodi, Syed Mudassir Qadri, Muneer Ahmad, Asrar Ahmad

**Affiliations:** *Department of Internal and Pulmonary Medicine, Sher-i-Kashmir Institute of Medical Sciences, Srinagar, Kashmir, India*; 1 *Department of Clinical Biochemistry, Sher-i-Kashmir Institute of Medical Sciences, Srinagar, Kashmir, India*

**Keywords:** Arterial, blood gases, COPD, correlation, pH, venous

## Abstract

**BACKGROUND:**

Arterial blood gas (ABG) analysis is routinely performed for sick patients but is fraught with complications, is painful, and is technically demanding.

**OBJECTIVE:**

To ascertain agreement between the arterial and peripheral venous measurement of pH, pCO_2_, pO_2_, and bicarbonate levels in sick patients with cardiopulmonary disorders in the valley of Kashmir in the Indian subcontinent, so as to use venous gas analysis instead of arterial for assessment of patients.

**SETTING:**

Sher-i-Kashmir Institute of Medical Sciences, Srinagar, Kashmir, a 650-bedded tertiary care hospital in North India located at an altitude of 1584 m.

**METHODS:**

One hundred patients who required ABG analysis were admitted. Peripheral venous blood was drawn within 5 min of an ABG measurement, and the samples analyzed immediately on a point of care automated ABG analyzer. Finger pulse oximetry was used to obtain oxygen (SpO_2_) saturation. Data were analyzed using Pearson correlation and bias (Bland Altman) methods.

**RESULTS:**

The venous measurements of pH, pCO_2_, pO_2_ and bicarbonate, and the digital oxygen saturation were highly correlated with their corresponding arterial measurements. Bland Altman plots demonstrated a high degree of agreement between the two corresponding sets of measurements with clinically acceptable differences. The difference in pO_2_ measurements was, however, higher (−22.34 ± 15.23) although the arterial saturation and finger oximetry revealed a good degree of agreement with clinically acceptable bias.

**CONCLUSION:**

Peripheral venous blood gas assessment in conjunction with finger pulse oximetry can obviate the routine use of arterial puncture in patients requiring ABG analysis.

Acid base analysis is an essential tool in acute care medicine, yielding valuable information about a diverse group of disorders. Arterial blood gas (ABG) is the gold standard method for acquiring this information. Although used to evaluate many respiratory and metabolic conditions, ABG analysis is not without drawbacks. Blood sample for ABG is obtained by arterial puncture. The risks associated with this procedure include pain, hematoma, aneurysm formation, thrombosis or embolization, needle stick injuries to health care providers, and sometimes even reflex sympathetic dystrophy.[1–4] The procedure is technically more demanding and is more painful. Over the years researchers have searched for alternatives to ABG sampling. Recently, studies have shown that values of acid–base balance measured in central or peripheral venous blood correlate well with those measured in arterial blood, at least for values of pH, bicarbonate, and carbon dioxide tension.[5–8] Venous blood is easier to obtain, the procedure being less painful, and there is an advantage of blood being drawn at a time when the patient is getting sampled for other investigations. While there have been few reports from different regions of the world, no study has specifically sampled cardiopulmonary patients from our part of world situated at a moderately high altitude of 1584 m where lung disorders form one of the commonest causes of hospital admissions, especially during the winter months.[9] As a result there is a huge requirement of performing ABG analysis. An alternative to the arterial puncture would be quite helpful in this setting, and the current study is aimed to determine the extent of correlation of arterial and venous blood gas (VBG) analysis with a view to identifying whether the venous samples can be used as an alternative to arterial values in the clinical management of patients presenting with various cardiopulmonary disorders.

## Methods

This prospective study was conducted in the Department of Internal and Pulmonary Medicine of Sher-i-Kashmir Institute of Medical Sciences, a 650-bedded tertiary care university hospital facility in the Northern Indian State of Jammu and Kashmir. A total of 100 randomly selected patients, who were adjudged to require ABG analysis by the treating physician, were enrolled. The patients included 63 cases admitted with chronic obstructive pulmonary disease (COPD) and exacerbation, 20 cases with community acquired pneumonia/sepsis, 11 cases with congestive heart failure, 5 cases with interstitial lung disease, 3 cases with post-tubercular fibrosis, and 1 patient with obstructive sleep apnea. All patients had arterial blood sampled which was drawn *via* an arterial puncture into a heparinized syringe. Simultaneously, venous blood in another heparinized syringe was also sampled *via* antecubital vein without application of any tourniquet. Venous and arterial blood samples were taken within 5-min of each other. In addition, the measurement of oxygen saturation (SpO_2_) was obtained from a finger pulse oximeter (Nonin pulse oximeter). Blood was analyzed immediately for values of acid-base, oxygenation and electrolyte status. The ABG and VBG analyzes were run on an immunoradiometric assay point-of-care blood gas analyzer that is operated by the Department of Biochemistry (Synthesis IL-45 analyzer, Instrumentation Laboratory, Lexington, MA). The assay is routinely used for analyzing blood gases in patients, and its accuracy is validated daily.

All the documented data were analyzed using Med Cal Statistical Software (version 10.4). The mean and 95% confidence intervals were calculated for each arterial and venous variable and for difference between them. The strength of the relationship between the arterial and venous gas values was assessed with the Pearson product–moment correlation coefficient test. The Bland–Altman limits of agreement were determined by plotting the difference between two paired values (arterial and venous) against their mean, thus creating a bias plot. The 95% limits of agreement represents the mean difference between each pair of venous and arterial values ±1.96 SD, and they estimate by how much a venous value is likely to differ from the criterion standard, the arterial value. This is the accepted method for assessing the agreement between two tests and represents a clinically relevant measure of comparison.[10,11] A *P* value of <0.05 for means and Pearson *r* was considered significant. Informed consent for participation in the study was obtained from all the participants, and the study was approved by the Institute Postgraduate and Ethics Committee.

## Results

A total of 100 patients who required ABG analysis were enrolled in this study, and 200 sets of blood gas values were analyzed. The mean age of the subjects was 44 ± 19.63 and the patients included 63 males and 37 females. Table 1depicts the mean, SD, and range of the values of arterial pH, venous pH, arterial paCO_2_, venous pCO_2_, arterial and venous bicarbonate, and arterial and venous pO_2_. In addition, the oxygen saturation as determined by ABG analysis (SaO_2_) and by digital pulse oximetry (SpO_2_) is depicted in Table 1. Arterial pH and venous pH were found to be correlated significantly (Pearson correlation coefficient *r* = 0.88, 95% confidence limits of *r* = 0.82-0.92, *P* < 0.001). There was a strong correlation between the arterial and venous pCO_2_(*r* = 0.92, 95% confidence limits of *r* = 0.88-0.94, *P* < 0.0001), bicarbonate values (*r* = 0.32, 95% confidence limits 0.13-0.49, *P* = 0.0012), and pO_2_ levels (*r* = 0.45, 95% confidence limits of *r* = 0.27-0.59, *P* < 0.0001). A significant correlation was also observed between SaO_2_ and SpO_2_(*r* = 0.59, 95% confidence limits of *r* = 0.45-0.71, *P* < 0.001).

**Table 1:** Summary statistics showing mean, standard deviation (SD), 95% confidence limits of mean (CI), median, and range of the various studied parameters

Parameter	Mean (SD)	95% CI	Median	Range
pH (Arterial)	7.46 (0.56)	7.45–7.47	7.47	7.257–7.59
pH (Venous)	7.43 (0.06)	7.42–7.44	7.43	7.176–7.58
pCO_2_ (Arterial, mmHg)	37.77*	35.88–39.75*	36.35*	20.70–71.00
pCO_2_ (Venous, mmHg)	41.69*	39.60–43.89*	40.10*	26.00–74.00
pO_2_ (Arterial, mmHg)	60.69 (16.63)	57.39–63.99	60.50	16.00–114.0
pO_2_ (Venous, mmHg)	38.35 (10.66)	36.23–40.46	38.00	13.00–75.00
HCO_3_ (Arterial mmol/l)	27.98 (6.23)	26.74–29.21	26.7	9.30–45.80
HCO_3_ (venous, mmol/l)	28.84 (6.88)	27.48–30.2	27.8	12.1–52.7
SaO_2_ (%)	89.08 (10.89)	86.9–91.27	92.6	21.7–99.0
SpO_2_ (%)	90.53 (7.31)	89.08–91.98	92.5	56.0–99.0

* Data not distributed normally have been back transformed after logarithmic transformation

**Figure 1:**
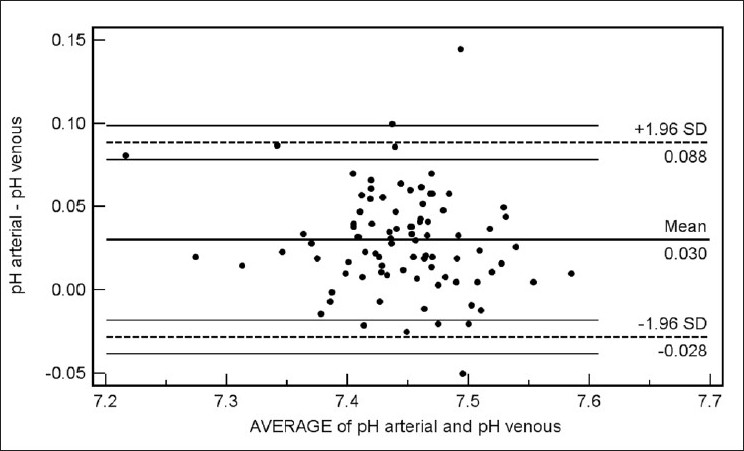
Bland Altman plots of arterial and venous pH (Average versus Difference)

**Figure 2:**
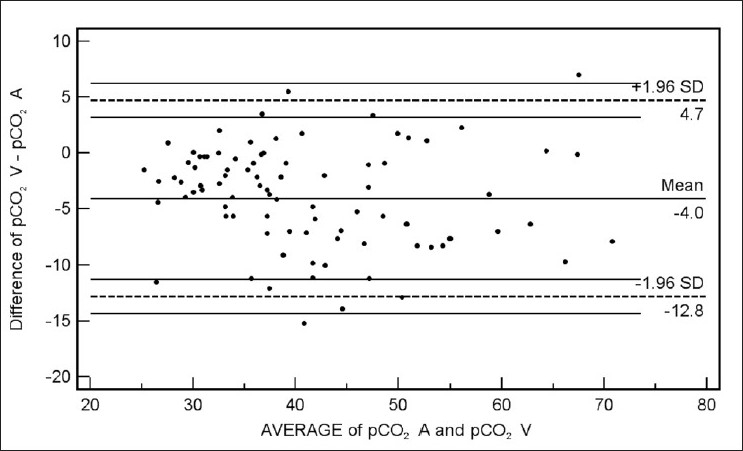
Bland Altman plots of arterial (pCO_2_ A) and venous pCO_2_ (pCO_2_ V) (Average vs. Difference)

**Figure 3:**
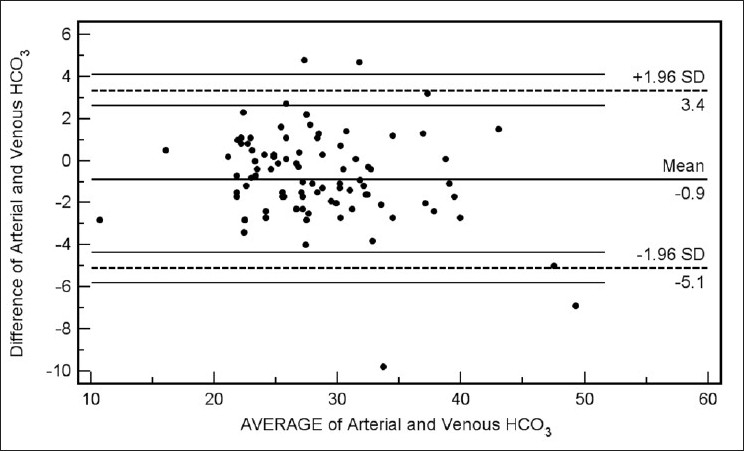
Bland Altman plots of arterial and venous HCO_3_ values (Average vs. Difference)

**Figure 4:**
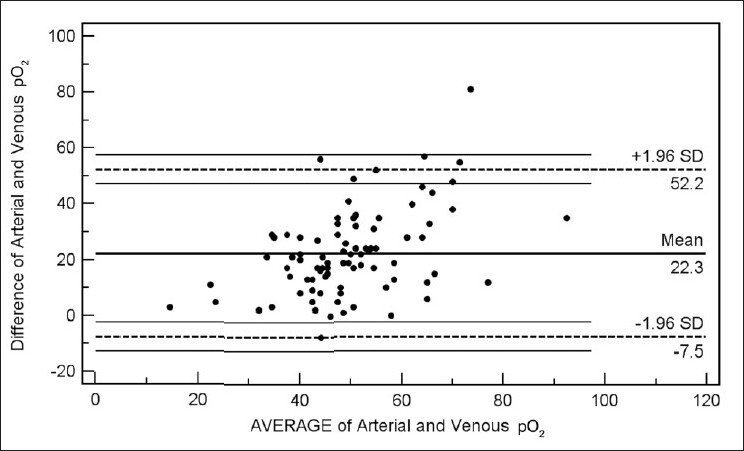
Bland Altman plots of arterial and venous pO_2_ (Average vs. Difference)

**Figure 5:**
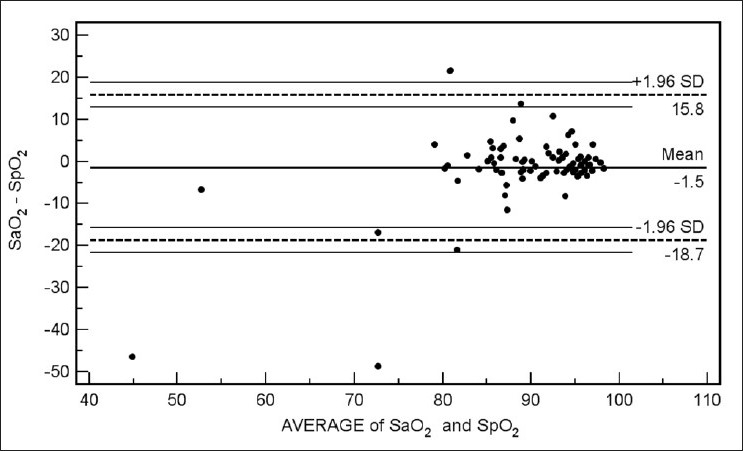
Bland Altman plots of measured oxygen saturation (SaO_2_) and on pulse oximetry (SpO_2_) (Average vs. Difference)

Bland Altman bias plots of the average of the arterial and venous measurements, and the difference between them are depicted in Figures 1–5. The bias plot for each of the variables showed excellent agreement with 95% limits of agreement being in acceptably narrow range in case of all the parameters. The mean bias in pH was –0.030 (SD = 0.030, 95% limits of agreement = –0.088 to 0.028); bias in pCO_2_ was –4.05 (SD = 4.48, 95% limits of agreement = –12.8 to 4.7), and in bicarbonate levels 086 (SD = 2.15, 95% limits of agreement = –3.35 to 5.09). Although the arterial and venous pO_2_ were significantly correlated with each other (*r* = 0.45, *P* < 0.0001), the bias between the two was –22.34 (SD = 15.23, 95% limits of agreement = –52.2 to 7.5). However, the bias between measured oxygen saturation (SaO_2_) and digital pulse oximetry (SpO_2_) was low (–1.44, SD = 8.82, 95% limits of agreement = –18.7 to 15.8), these results thus demonstrate an acceptably low bias with excellent agreement between the arterial and peripheral venous values.

## Discussion

Our data have demonstrated a high level of correlation between the arterial and venous measurements of pH, pCO_2_, and bicarbonate level with a high degree of agreement and clinically acceptable difference on Bland Altman difference plotting. Although there is a significant correlation between the arterial and venous measurements of pO_2_ level, the difference of -22.34 ± 15.23 is not clinically acceptable enough to support uniform usage of venous pO_2_ instead of arterial measurements in clinical situations. However, SaO_2_ and SpO_2_ were highly correlated and demonstrated excellent agreement and a clinically acceptable difference (-1.44 ± 8.82). Caution is required for using correlation coefficients to describe the agreement between two quantitative variables. A high correlation is definitely required but does not necessarily imply agreement, for which the line of agreement should be a 45° (*x* = *y*) line. Bland Altman plots are used instead to describe the agreement between two quantitative variables, the difference of the paired two observations plotted against the mean of two measurements and 95% of the data points recommended to lie within ±2D of the mean difference.[12]

Several researchers have looked at the possibility of using the venous or capillary data instead of ABGs and have reported differing results. Gambino compared “arterialized” capillary blood with arterial samples from the brachial artery in patients undergoing pulmonary function tests and found no difference in pH and CO_2_ content.[13] Subsequently Zahn and Weil[14] examined the relationship between the arterial and central venous pH and concluded that central venous pH was a reliable indicator of arterial pH. However, Adrogue *et al*.[15] emphasized that both arterial and central venous sampling are needed in patients with critical hemodynamic compromise. Other investigators have also supported the use of venous measurements instead of arterial measurements for pH and gas analysis[16,17] while others[18] have resisted the idea. Arterial or venous data have been rarely noted to influence the physician’s treatment in emergency in case of diabetic ketoacidosis,[16,19] and venous values have been demonstrated to have excellent agreement with arterial values in cases of uremic acidosis.[20]

All the samples of the patients in our study were taken in the supine position. In a study comparing the effect of various body positions on the ABGs, there were essentially no significant differences in pO_2_ among supine, sitting, and standing positions in asymptomatic patients of different age-groups or in patients with COAD[21] even as another study demonstrated that because of unpredictable postural changes in PaO_2_ in patients with COPD, body position should be noted for ABG measurements and should be kept constant for valid comparison of serial measurements.[22]

The majority of our patients had lung disorders with COPD forming the commonest condition. Earlier COPD patients on mechanical ventilation[23] and those managed without mechanical ventilation[12] have been studied and both the groups of investigators reported that VBG measurements could be used instead of ABG analysis for assessment of the patients. The patients included in our study resided at a moderately high altitude. It is known that the barometric pressure decreases with distance above the Earth’s surface in an approximately exponential manner. The pressure at 5500 is only about one-half the normal 760 mmHg, so the pO_2_ of moist inspired gas is (380-47) + 0.2093 = 70 mmHg (partial pressure of water vapor equals 47 mmHg). In spite of hypoxia associated with high altitude, some 15 million people live at elevations over 3050 m depicting a remarkable degree of acclimatization at these altitudes. The permanent residents who live at these altitudes have a degree of hyperventilation that compensates for the degree of hypoxia, but at the expense of decreased PCO_2_. The hyperventilation results from the hypoxic stimulation of the peripheral chemoreceptors. Graham and Houston[24] have reported that patients with COPD (without CO_2_ retention) tolerate high altitudes of up to 2000 m well, complaining of fatigue only.[24,25] This explains the behavior of our patients at the moderate altitudes of Kashmir. Whether altitude of this magnitude plays a role in the blood gases needs to be studied in greater detail subsequently. However in the context of the current study, even if does, it would presumably affect the arterial and venous gases similarly.

We used peripheral venous blood in our study for VBG assessment. While analysis of central venous blood has been previously used for data comparison of VBG versus ABG[5,15,26] sampled in central venous catheters in ICU patients, the sampling of peripheral blood allows greater flexibility in obtaining data in patients without central venous access. Our results fortify the sparse data[5,7,27] regarding the view that peripheral VBG analysis can be used for fairly accurate assessment of arterial pH, pCO_2_, and bicarbonate values with clinically acceptable differences between the arterial and the venous values. Although there is a high correlation between the arterial and venous pO_2_ values, the difference is large for total clinical agreement and thus suggesting that one cannot be swapped for the other without loss of important detail. However, SaO_2_ and SpO_2_ have high degree of correlation and high degree of agreement with clinically acceptable difference. Thus if venous pH is combined with routine finger pulse oximetry, the ABG analysis might be minimized to a great degree in cardiopulmonary patients. Additional studies may be necessary to address the feasibility and reproducibility of such testing.
